# Estrone Sulfate Transport and Steroid Sulfatase Activity in Colorectal Cancer: Implications for Hormone Replacement Therapy

**DOI:** 10.3389/fphar.2017.00103

**Published:** 2017-03-07

**Authors:** Lorna C. Gilligan, Ali Gondal, Vivien Tang, Maryam T. Hussain, Anastasia Arvaniti, Anne-Marie Hewitt, Paul A. Foster

**Affiliations:** ^1^Institute of Metabolism and Systems Research, Centre for Endocrinology, Diabetes, and Metabolism, University of BirminghamBirmingham, UK; ^2^Centre for Endocrinology, Diabetes, and Metabolism, Birmingham Health PartnersBirmingham, UK

**Keywords:** estrogen, colorectal cancer, steroid sulfatase, OATP, SLCO, GPER, tamoxifen

## Abstract

Hormone replacement therapy (HRT) affects the incidence and potential progression of colorectal cancer (CRC). As HRT primarily consists of estrone sulfate (E_1_S), understanding whether this conjugated estrogen is transported and metabolized in CRC will define its potential effect in this malignancy. Here, we show that a panel of CRC cell lines (Colo205, Caco2, HCT116, HT-29) have steroid sulfatase (STS) activity, and thus can hydrolyze E_1_S. STS activity is significantly higher in CRC cell lysate, suggesting the importance of E_1_S transport in intracellular STS substrate availability. As E_1_S transport is regulated by the expression pattern of certain solute carrier organic anion transporter polypeptides, we show that in CRC OATP4A1 is the most abundantly expressed transporter. All four CRC cell lines rapidly transported E_1_S into cells, with this effect significantly inhibited by the competitive OATP inhibitor BSP. Transient knockdown of OATP4A1 significantly disrupted E_1_S uptake. Examination of estrogen receptor status showed ERα was present in Colo205 and Caco2 cells. None of the cells expressed ERβ. Intriguingly, HCT116 and HT29 cells strongly expressed the G protein coupled estrogen receptor (GPER), and that stimulation of this receptor with estradiol (E_2_) and G1, a GPER agonist, significantly (*p* < 0.01) increased STS activity. Furthermore, tamoxifen and fulvestrant, known GPER agonist, also increased CRC STS activity, with this effect inhibited by the GPER antagonist G15. These results suggest that CRC can take up and hydrolyze E_1_S, and that subsequent GPER stimulation increases STS activity in a potentially novel positive feedback loop. As elevated STS expression is associated with poor prognosis in CRC, these results suggest HRT, tamoxifen and fulvestrant may negatively impact CRC patient outcomes.

## Introduction

Estrogens play an important role in the etiology of CRC (Foster, 2013). Pre-menopausal women are protected against CRC compared to age-matched men (Farquhar et al., 2005), and data from the Women’s Health Initiative suggests post-menopausal women on HRT, a combination of estrone sulfate (E_1_S) and progestins, have a 40% reduced incidence of developing CRC compared to women not on HRT (Chlebowski et al., 2004).

Despite this epidemiological evidence suggesting HRT as protective against CRC, evidence also exists showing estrogens influence CRC proliferation. For example, the CRC cell line Lovo increases proliferation, via a FASN-mediated mechanism, when exposed to estradiol (E_2_) (Santolla et al., 2012). Indeed, E_2_ increases the proliferation of the CRC cell line Caco2 (Di Domenico et al., 1996) and T84 (Hennessy et al., 2005), and inhibits apoptosis in DLD-1 cells (Messa et al., 2005). CRC patients on HRT present at a later and more advanced stage of disease (Chlebowski et al., 2004), suggesting estrogens as mitogenic in CRC. Thus, the ability of CRC to take up and consequently metabolize E_1_S will define local concentrations of active estrogens and subsequent action.

**FIGURE 1:**
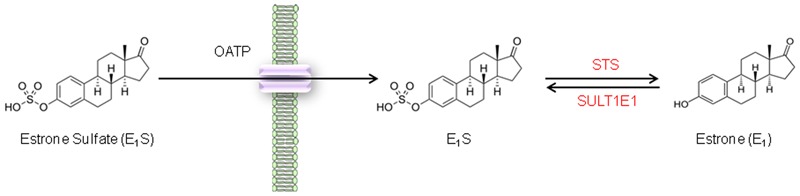
**Steroid sulfatase activity in CRC cell lines.** E_1_S is transported across the cell membrane by OATPs and once intracellular E_1_S can be hydrolyzed by STS to form E_1_.

Steroid sulfatase is the key enzyme involved in hydrolyzing E_1_S to E_1_ (**Figure 1**) (Mueller et al., 2015), and its activity is known to directly increase the proliferation of estrogen-dependent breast cancer (Foster et al., 2006) and endometrial cancer (Foster et al., 2008b). Inhibition of STS has shown significant promise against ERα positive breast cancer (Stanway et al., 2006; Foster et al., 2008a). Intriguingly, STS expression, elevated E_1_ and E_2_ intratumoural concentrations, are associated with a poor prognosis in CRC patients (Sato et al., 2009). This suggests that colonic estrogen metabolism may play an important role in CRC patient outcomes.

However, very little is known about whether E_1_S is transported into CRC. Sulfated steroids require transport into cells via solute carrier organic anion transporting polypeptides (SLCO/OATP); membrane bound proteins that transport a plethora of organic anions (Roth et al., 2012). Six different SLCO/OATP (OATP1A2, OATP1B1, OATP1B3, OATP2B1, OATP3A1, OATP4A1) are known to effectively transport E_1_S (Mueller et al., 2015), although their expression and action in CRC is poorly defined. Other OATPs are known to transport E_1_S (OATP4C1 and OATP1C1), however OATP4C1 is primarily expressed in the kidney (Mikkaichi et al., 2004), and microarray analysis shows it may have some expression in the liver but not the human colon (Bleasby et al., 2006). OATP1C1 is localized to human brain and testis (Pizzagalli et al., 2002), and is not evident in human colorectal tissue, as measure by microarray (Bleasby et al., 2006).

Here, we investigate STS activity and E_1_S transport kinetics in four CRC cell lines. We demonstrate that OATP4A1 is most likely the principle E_1_S transporter in CRC and that all cell lines have the ability to hydrolyze E_1_S. We also show that STS activity in CRC may be regulated by local E_2_ availability via a novel GPER mechanism.

## Materials and Methods

### Compounds

STX64 (Irosustat, 667COUMATE) was kindly supplied by Prof. Barry Potter (University of Oxford, UK). G1 and G15 were purchased from Torcis Bioscience (Abingdon, UK). E_2_, tamoxifen, fulvestrant, and BSP were purchased from Sigma-Aldrich (Dorset, UK).

### Cell Culture

The CRC cell lines Colo205, HCT116, and HT29 were purchased from the American Type Culture Collection, USA; Caco2 and JEG3 cells were purchased from The European Collection of Cell Cultures (ECACC). Prior to experiments, all cell lines were authenticated by short tandem repeat profiling and were used between passages 10 and 35. Furthermore, all experiments were performed during the exponential growth phase of the cell line. HCT116 and HT29 were routinely cultured in McCoy’s 5a modified medium (Gibco, Life Technologies, USA) with 10% v/v heat inactivated FBS (Sigma-Aldrich, UK). Colo205 cells were culture in RPMI with 10% FBS; Caco2 cells were cultured in MEM with 10% FBS. JEG3 cells were cultured in DM-F12 (Gibco, USA) with 10% FBS. All culture mediums were supplemented with 2 mM L-glutamine (Sigma-Aldrich, UK) and 1% PenStrep (Gibco, USA). JEG3 cells were used as control as they exhibit high STS activity.

For experimental conditions, cells were initially starved of estrogens for 72 h by placing them in their appropriate phenol-red free medium plus 10% charcoal stripped FBS (sFBS) (Sigma-Aldrich, UK). After starvation, HCT116 and HT29 cells were treated with E_2_ (100 nM), G1 (100 nM), G15 (1 μM), Tamoxifen (10 nM) or fulvestrant (1 μM) in stripped medium for 24 h prior to measuring STS activity.

### STS Activity Assay

*In vitro* STS activities of cell lines were measured as previously described (Purohit et al., 1997). Briefly, in intact cell assays, cells were incubated with appropriate medium containing [6,7-^3^H] E_1_S (4 × 10^5^ dpm, Perkin-ElmerLS, Boston, MA, USA) adjusted to a final concentration of 20 μM with unlabeled E_1_S (Sigma-Aldrich, UK). [4-^14^C] E_1_ (1 × 10^4^ dpm, Perkin-Elmer) was also included in the reaction mixture to monitor procedural losses. Samples were incubated for 18 h at 37°C after which the product, E_1_, was separated from E_1_S by partition with toluene. A toluene aliquot was removed and ^3^H and ^14^C radioactivity measured by liquid scintillation spectrometry. The mass of E_1_S hydrolyzed was calculated from the ^3^H counts detected corrected for procedural losses. A protein measurement was obtained for the cells by lysing the cells with RIPA buffer (Sigma-Aldrich, UK) followed by a BCA assay (Thermo Fisher Scientific, UK).

To determine STS activity in cell lysates, cells were first lysed with RIPA buffer and protein content determined using a BCA assay. Subsequently, 100 μg of cell protein was incubated for 4 h with PBS containing [6,7-^3^H] E_1_S (4 × 10^5^ dpm) adjusted to a final concentration of 20 μM with unlabeled E_1_S. [4-^14^C] E_1_ (1 × 10^4^ dpm) was again used to monitor procedural losses. E_1_ was separated from E_1_S by partition with toluene and ^3^H and ^14^C radioactivity measured by liquid scintillation spectrometry. Results for both intact and cell lysates were determined as pmol E_1_ formed/h/mg protein.

### qRT-PCR Analysis

From cells mRNA was purified from T75 flasks or six well plates at approximately 80% confluency using RNeasy kits (QIAGEN, Crawley, UK) and stored at -80°C. Aliquots containing 5 μg of mRNA were reverse transcribed in a final volume of 20 μl to form cDNA using Tetro cDNA Synthesis Kit (Bioline Reagents, Ltd, UK). RT-PCR reactions were performed in a ‘Rotor Gene 2000 Real-Time Cycler’ (Corbett Life Science, Cambridge, UK) with 1 μl cDNA in a final volume of 12 μl, using Taqman universal PCR master mix and Taqman expression assays containing primers and probes for OATP1A2, OATP1B1, OATP1B3, OATP2B1, OATP3A1, OATP4A1, and for the endogenous control gene, RPLP0 (Applied Biosystems, UK). In CRC, RPLPO is considered the most reliable single standard gene to examine (Sørby et al., 2010). The conditions were as follows: 95°C for 10 min; followed by 40 cycles of 95°C for 15 s, and 60°C for 60 s. Relative mRNA expression was calculated using the comparative quantisation algorithm in the Rotor Gene 6 software (Corbett Life Science).

### E_1_S Uptake Studies

Cells were seeded at 200,000 cells per well in 6-well plates and allowed to acclimatize for 24 h. Appropriate phenol red free medium containing 10% sFBS plus [6,7-^3^H] E_1_S (4 × 10^5^ dpm) was placed on the cells and was subsequently removed after 2, 5, 10, 15, 20, 30 min. For OATP inhibition studies, the competitive OATP inhibitor BSP (at 1 μM) was added to the medium and therefore was present throughout the 30 min uptake time. The cells were then washed twice in PBS, lysed using RIPA buffer, and the intracellular ^3^H radioactivity measured by liquid scintillation spectrometry. Cell protein content was also determined with a BCA assay. Results are expressed as E_1_S uptake pmol/mg protein.

### Immunoblotting

Protein concentration was determined from cell lysates using the BCA assay, and 15 μg samples were separated by electrophoresis under reducing conditions on 4–12% Bis-Tris 10% SDS-PAGE gels (Invitrogen, Paisley, UK) before being transferred to PVDF membranes. Membranes were immunoblotted with either ERα (1:1000), ERβ (1:1000), GPER (1:800) (all from Santa Cruz, UK), or β-actin (1:50,000) (Sigma-Aldrich, UK) monoclonal antibodies in incubation buffer containing 0.1% milk (Marvel; Premier Brands UK Ltd, Lincolnshire, UK) in TBST. For full details of antibodies and conditions used see **Table 1**. Bound antibody was detected with horseradish peroxidase-conjugated anti-mouse or anti-rabbit secondary antibodies and chemiluminescence detection.

**Table 1:** Information regarding antibodies and conditions used for immunoblotting.

Antibody	Manufacturer, Cat. #, Lot #	Peptide/protein target	Species raised, monoclonal/polyclonal	Dilution used	Positive control
ERα	Santa Cruz, sc-130072, C2910	aa 301–595	Mouse, monoclonal	1:1000, 1% milk	MCF-7
ERβ	Abcam, ab288, GR79420-5	aa 1–153	Mouse, monoclonal	1:1000, 1% milk	MCF-7
GPER	Santa Cruz, sc-48525-R, F2414	N-terminus	Rabbit, Polyclonal	1:800, 5% milk	MCF-7
β-actin	Sigma, A228	slightly modified β-cytoplasmic actin N-terminal peptide	Mouse, Monoclonal	1:50,000	

### siRNA Transfection

Twenty four hours after seeding HCT116 cultures were transfected with OATP-specific and control siRNA (Thermo Fisher Scientific, UK) by lipofectamine-2000 (Invitrogen) using standard protocols. 72 h post-transfection, E_1_S uptake studies were performed over 30 min and total E_1_S uptake calculated.

## Results

### STS Activity in Intact and Lysed CRC Cell Lines

**FIGURE 2:**
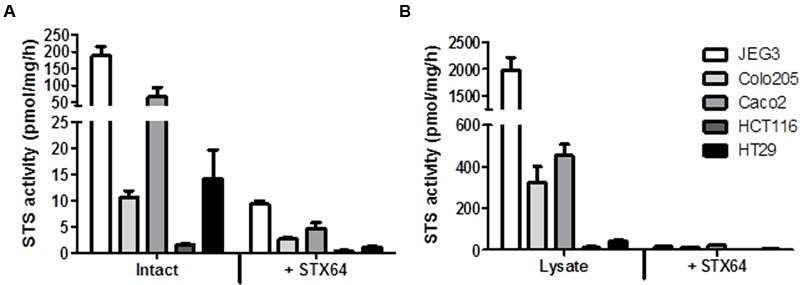
**(A)** Steroid sulfatase activity (E_1_S conversion in pmol/mg/h) and inhibition by STX64 (1 μM) in intact CRC cell lines. **(B)** STS activity and inhibition by STX64 (1 μM) in CRC cell lysate. JEG3 cells were used as a positive control in all experiments. Data represents mean ± SD. *n* = 3 independent experiments.

The panel of four CRC cell lines, plus the positive control JEG3, were investigated and demonstrate a wide range of STS activity (**Figures 2A,B**). In intact (not lysed) cells, Caco2 exhibited the highest STS activity (65.47 ± 28.51 pmol/mg/h), with HCT116 cells having the lowest activity (1.65 ± 0.14 pmol/mg/h). STS activity could be almost completely inhibited in intact cells using 1 μM of the specific STS inhibitor STX64 (**Figure 2A**).

When lysed, the intracellular STS activity was much greater in all cell lines examined compared to their intact state (**Figure 2B**). STS activity was again greatest in the Caco2 cell lines (452.90 ± 56.34 pmol/mg/h) with HCT116 cell showing the lowest STS activity (11.87 ± 6.41 pmol/mg/h). These results demonstrate that E_1_S uptake kinetics most likely dictate the ability of CRC to hydrolyze E_1_. Therefore, as E_1_S is transported through the cell membrane by OATPs the expression of these transporters was next determined.

### OATP Expression and E_1_S Uptake in CRC Cell Lines

**FIGURE 3:**
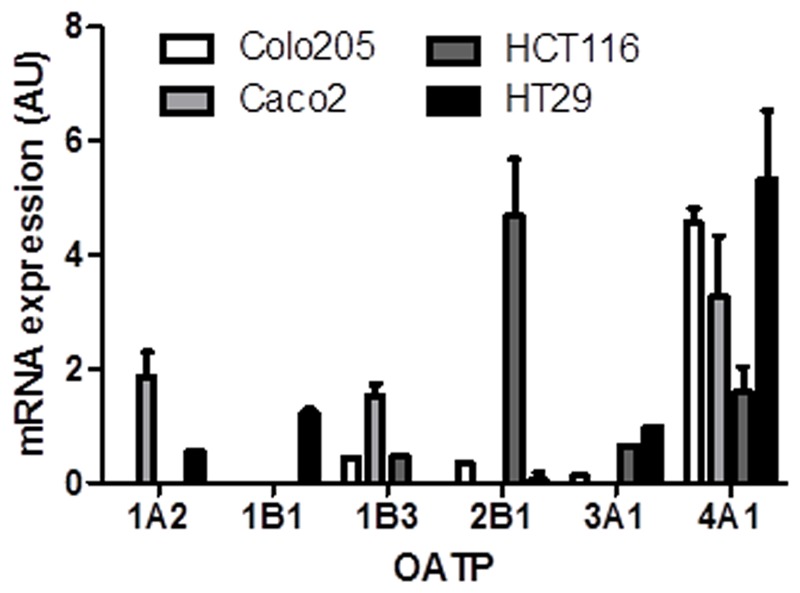
**Organic anion transporter polypeptide mRNA expression in Colo205, Caco2, HCT116, and HT-29 cells.** OATP4A1 expression was the highest in all cell lines. Data represents mean ± SD. *n* = 3 independent experiments.

The mRNA expression of OATPs known to transport E_1_S across the plasma membrane was determined in the panel of CRC cell lines (**Figure 3**). Of the six OATPs examined only OATP4A1 was present in all four cell lines. OATP2B1 expression was also notably high in Caco2 cells. The HT29 cells expressed five of the six OATPs determined (OATP1B3 was not present in HT29 cells), with the Caco2 cells expressing four (OATP1A1, OATP1B3, OATP2B1, OATP4A1) out of six. Our results are roughly consistent with the expression patterns obtained from the CellMiner database^1^, as shown by average transcript log^2^ intensities (see **Table 2**).

**Table 2:** Average transcript log^2^ intensities determined from the National Cancer Institute (NCI) CellMiner database (https://discover.nci.nih.gov/cellminer/).

	1A2	1B1	1B3	2B1	3A1	4A1
Colo205	3.24	3.22	5.50	7.29	4.76	8.38
HCT116	2.96	3.23	5.75	5.34	4.61	8.19
HT29	2.84	2.89	4.92	5.39	6.54	8.04

*Average log^2^ determined from mRNA various probes/probe sets.*

**FIGURE 4:**
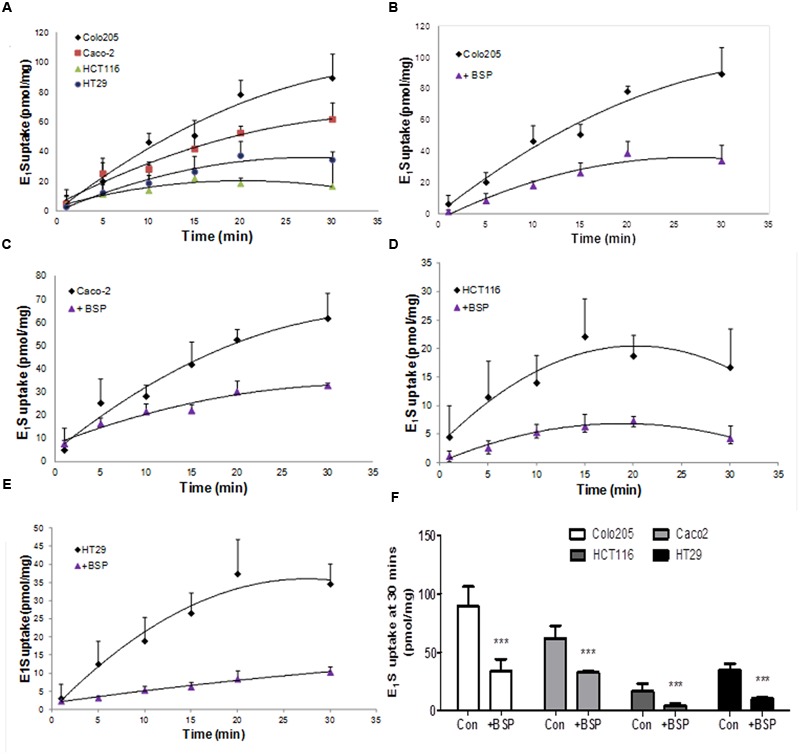
**Estrone sulfate transport in Colo205, Caco2, HCT116 and HT-29 cells. (A)** Comparison of the different uptake kinetics of E_1_S over 30 min in four CRC cell lines. **(B)** Inhibition of Colo205 E_1_S transport by BSP (1 mM). **(C)** Inhibition of Caco2 E_1_S transport by BSP. **(D)** Inhibition of HCT116 E_1_S transport by BSP. **(E)** Inhibition of HT-29 E_1_S transport by BSP. **(F)** Total E_1_S uptake over 30 min by four CRC cell lines and the total inhibition caused by BSP. Data represents mean ± SD. *n* = 3 independent experiments. ^∗∗∗^*p* < 0.001 compared to control.

When the ability of CRC cells to transport E_1_S into the cells was determined, Colo205 demonstrated the quickest uptake rate, with HCT116 cells having the slowest uptake (**Figure 4A**). Inhibition of OATP transport with the non-specific OATP inhibitor BSP significantly reduced E_1_S uptake in all four cell lines (**Figures 4B–E**). Caco2 cells had the most rapid with HCT116 cells exhibited the slowest E_1_S transport. E_1_S uptake after 30 min demonstrated that Colo205 (89.41 ± 16.80 pmol/mg) and Caco2 (61.78 ± 10.80 pmol/mg) cells exhibited the most intracellular E_1_S transport, with HCT116 (16.73 ± 6.80 pmol/mg) and HT29 (34.59 ± 5.63 pmol/mg) cells showing lower E_1_S transport (**Figure 4F**). BSP significantly (*p* < 0.001) inhibited E_1_S transport in all four cell lines.

**FIGURE 5:**
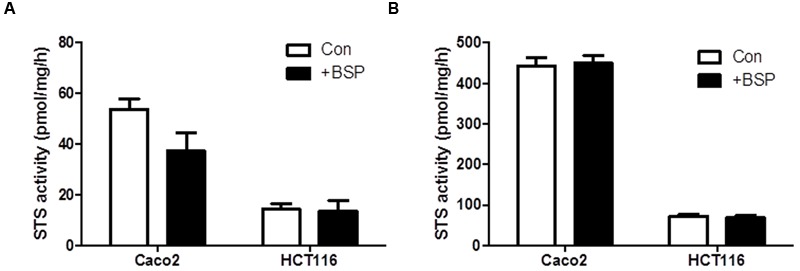
**Bromsulfthalein has limited effect on STS activity Caco2 and HCT116 cells. (A)** In intact cells, STS activity is non-significantly reduced by BSP (1 μM) in Caco2 cells and has no effect on HCT116 cells. **(B)** In cell lysates, BSP (1 μM) has no effect on STS activity in Caco2 or HCT116 lysates. Data represents mean ± SD. *n* = 3 independent experiments.

To examine if OATP inhibition by BSP reduces E_1_S intracellular substrate availability to STS, we examined the effect of BSP on STS activity in intact cells and cell lysates. We selected Caco2 and HCT116 cells as representative of CRC cells with high and low STS activity, respectively. In intact cells, treatment with BPS (1 μM) non-significantly reduced STS activity in Caco2 cells but had no effect on HCT116 STS activity (**Figure 5A**). When BPS (1 μM) was tested in cell lysates it had no effect on STS activity in both Caco2 and HCT116 cells, suggesting it does not directly inhibit the STS enzyme (**Figure 5B**). These results imply that OATP transport into cells plays a rate-limiting step on E_1_S STS hydrolysis. The discrepancy between Caco2 and HCT116 cells in response to BSP most likely represents the difference between the STS activity in the cell lines. When STS activity is high (Caco2), limiting E_1_S OATP transport limits intracellular E_1_S availability thus reducing E_1_ hydrolysis. When STS activity is low (HCT116), limiting E_1_S OATP transport does not directly translate to decreased hydrolysis as there is less STS activity, and thus limiting substrate availability via OATP inhibition and where enzyme activity is already low does not translate to reduced E_1_S hydrolysis.

**FIGURE 6:**
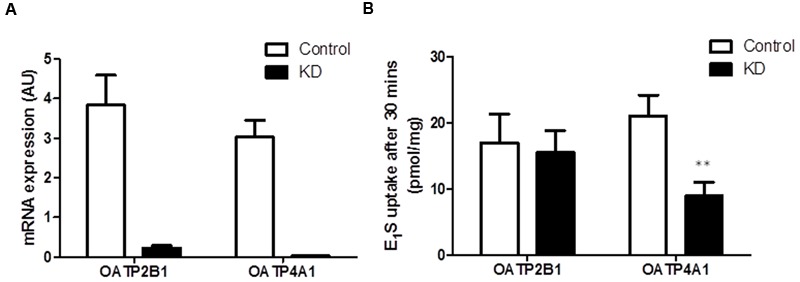
**OATP4A1 knockdown in HCT116 cells blocks E_1_S transport. (A)** siRNA knockdown of OATP2B1 and OATP4A1 reduced transporter expression by over 95%. **(B)** In HCT116 cells OATP2B1 knockdown has no effect on E_1_S transport, however OATP4A1 knockdown inhibited E_1_S transport by 52.4%. Data represents mean ± SD. *n* = 3 independent experiments. ^∗∗^*p* < 0.01 compared to control.

As OATP2B1 and OATP4A1 were expressed at the highest concentrations compared to the other OATPs, we next performed siRNA knockdown of these two transporters in HCT116 cells to determine their importance on E_1_S uptake. We used HCT116 cells as they had high OATP2B1 and OATP4A1 expression without high expression of any other OATPs. Furthermore, HCT116 cells are readily transfected by lipofectamine for siRNA delivery. For both OATPs siRNA gave < 95% knockdown as measured by qRT-PCR (**Figure 6A**). OATP2B1 knockdown did not significantly affect E_1_S uptake (**Figure 6B**), however OATP4A1 knockdown significantly reduced E_1_S uptake from 21.02 ± 3.12 to 8.93 ± 2.07 pmol/mg/h (*p* < 0.01), suggesting this transporter may play a key role in E_1_S transport in CRC.

### STS Activity is Regulated by E_2_ Availability and GPER Action in CRC

**FIGURE 7:**
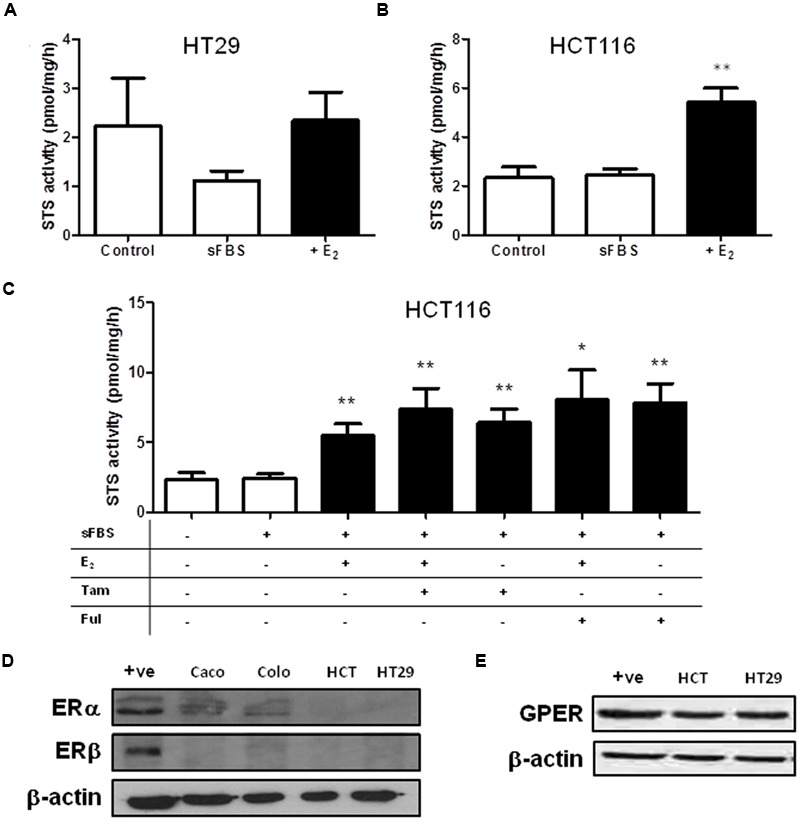
**Steroid sulfatase activity is regulated by estrogen availability in CRC cell lines. (A)** E_2_ (100 nM) does not increase STS activity in HT29. **(B)** E_2_ (100 nM) significantly increase STS activity in HCT116 cells. **(C)** 24 h treatment of Tamoxifen (10 nM) and fulvestrant (1 mM) increases STS activity in HCT116 cells. **(D)** Colo205 and Caco2 cells express ERα, but not ERβ. HCT116 and HT29 cells do not express ERα or ERβ. The +ive control is MCF-7 protein. **(E)** GPER is expressed in HCT116 and HT29 cells. Data represents mean ± SD. *n* = 3 independent experiments. ^∗^*p* < 0.05, ^∗∗^*p* < 0.01 compared to sFBS control.

As steroid metabolism and estrogen action in the colon is poorly defined we next speculated whether estrogens may influence steroid sulfatase activity, as it has been reported to do in other malignancies (Zaichuk et al., 2007). Thus we next examined how E_2_ starvation and subsequent E_2_ supplementation effects STS activity. HT29 cell treated with sFBS medium (i.e., estrogen starvation) demonstrated a trend toward reduction in STS activity, with this effect reversed when supplemented with E_2_ (**Figure 7A**). In HCT116 cells, 24 h of E_2_ (100 nM) treatment significantly (*p* < 0.01) induced STS activity compared to sFBS controls (**Figure 7B**).

We next attempted to inhibit this E_2_-induced increase in STS activity by treating HCT116 with Tamoxifen or fulvestrant co-administered with E_2_. Surprisingly, both Tamoxifen and fulvestrant significantly increased STS activity in HCT116 cells (**Figure 7C**) and neither compound had any effect on E_2_-induced STS activity. Tamoxifen (at 10 nM) increased STS activity to 6.43 ± 0.95 pmol/mg/h and fulvestrant (1 μM) increase activity to 7.84 ± 1.36 pmol/mg/h compared to 2.43 ± 0.31 pmol/mg/h.

**FIGURE 8:**
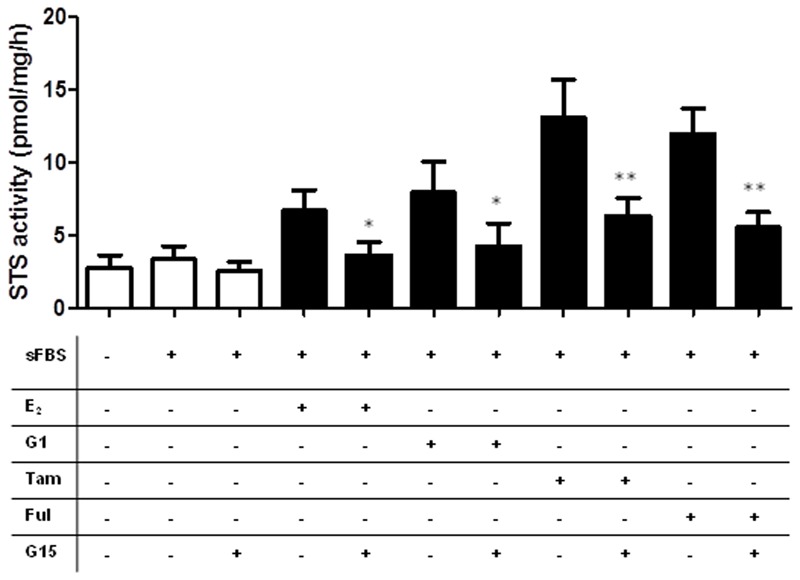
**Stimulation of GPER increases STS activity in HCT116 cells.** E_2_ (100 nM), G1 (100 nM), Tamoxifen (10 nM), and fulvestrant (1 mM) induce increased STS activity in HCT116 cells. These effects are inhibited by the addition of the GPER antagonist G15 (1 mM). Data represents mean ± SD. *n* = 3 independent experiments. ^∗^*p* < 0.05, ^∗∗^*p* < 0.01.

Examination of the CRC cell lines ER status demonstrated that ERα protein expression was present in Caco2 cells, and lowly expressed in Colo205 cells. HCT116 and HT29 cells did not express ERα (**Figure 7D**). None of the cell lines expressed ERβ (**Figure 7D**). We also assessed the GPER status in our CRC cell lines. HCT116 and HT29 cells expressed GPER (**Figure 7E**), as did Caco2 and Colo205 (data not shown). Unedited immunoblots are shown in Supplementary Figure 1. As both Tamoxifen and fulvastrant have been shown to be GPER agonist (Prossnitz and Barton, 2014) we next examined whether the increased STS activity induced by these compounds could be inhibited by G15, a specific GPER antagonist (Dennis et al., 2009). In HCT116 cells, G15 (1 μM) significantly inhibited the increase of STS activity induced by 24 h treatment with E_2_ (100 nM), the GPER agonist G1 (100 nM), Tamoxifen (10 nM), and fulvestrant (1 μM) (**Figure 8**). This suggests a potential novel positive estrogen metabolism feedback loop through GPER stimulation is present in CRC.

## Discussion

Hormone replacement therapy, usually a combination of equine E_1_S and progestins, may play a dual role in the incidence and progression of CRC. Initially protective against the development of CRC (Chlebowski et al., 2004), once the malignancy is formed CRC may be estrogen responsive (Foster, 2013). Thus, defining how CRC transports and metabolizes estrogens, and the ER status of CRC, may define how this tissue responds to HRT. Here we demonstrate that CRC cell lines possess STS activity (see **Figure 2**) and are able to transport E_1_S (see **Figure 4**), most likely through OATP4A1 (see **Figure 6**), into cells. Furthermore, we show that STS activity is elevated by local E_2_ concentrations (see **Figure 7**) via GPER action. Finally we show that tamoxifen and fulvestrant, both GPER agonists, also elevate STS activity (see **Figure 8**) indicating that its use in breast cancer patients may have unwanted consequences in the colon.

Elevated STS expression has been demonstrated in breast (Pasqualini et al., 1996), prostate (Nakamura et al., 2006) endometrial (Lepine et al., 2010), and epithelial ovarian cancer (Ren et al., 2015). We demonstrate here for the first time that STS activity is present in both intact and lysed CRC cells, and this activity could be inhibited with STX64 (**Figure 2**). Although the role of STS activity and estrogens in CRC is not yet defined, our results, combined with evidence showing increased STS expression as a poor prognostic indicator in CRC patients (Sato et al., 2009), suggests STX64 as a potential therapeutic option for this disease. Indeed, STX64 has shown significant promise in hormone-dependent breast cancer patients (Stanway et al., 2006, 2007) with limited adverse events (Stanway et al., 2006). Further Phase II trials of STX64 are currently ongoing in patients with hormone-dependent breast cancer.

Cell protein (from cell lysates) exhibited significantly greater STS activity compared to intact cells suggesting E_1_S cell membrane transport as the limiting factor in estrogen hydrolysis. Thus, OATP expression patterns in CRC may be a key regulator of E_1_S transport and subsequent estrogen action. Our results demonstrate OATP4A1, followed by OATP2B1, as the most abundantly expressed E_1_S transporters in CRC cell lines (**Figure 3**). OATP4A1 is up-regulated and hypomethylated in CRC compared to normal colon tissue (Rawluszko-Wieczorek et al., 2015), suggesting its importance in disease progression, and implying that E_1_S uptake through this transporter may influence tumor proliferation. Furthermore, OATP3A1 is down-regulated in CRC (Rawluszko-Wieczorek et al., 2015), and reflected in our results as low expression of this transporter is evident in all four cell lines. OATP1B3 correlates to the Gleason score as a marker of CRC dedifferentiation: higher OATP1B3 expression in the colon is associated with earlier tumor stage and improved tumor differentiation (Pressler et al., 2011). We demonstrate low OATP1B3 expression in our cell lines, supporting a role for this transporter in early tumor stage as all four cell lines examined were derived from latter-stage tumors.

Once transported, E_1_S requires hydrolysis to form E_1_, and subsequently E_2_ via 17β-hydroxysteroid dehydrogenase activity. However, controversy surrounds how estrogens act in CRC. Evidence suggests ERα is either lowly expressed (Cavallini et al., 2002) or not present (Witte et al., 2001), and ERβ is down-regulated during CRC development from colon adenomas (Konstantinopoulos et al., 2003). Thus, we determined ER status in four CRC cell lines, showing that Caco2 and Colo205 cells have some ERα immunoreactivity (**Figure 7**). None of the cell lines expressed ERβ. As they did not express either ERα or ERβ, we selected HCT116 and HT29 cells and determined that they expressed GPER. As ERα and ERβ are not present in CRC, this suggests that estrogens may primarily act through GPER in CRC. Indeed, estrogen binding to GPER increases colonic transit time (Li et al., 2016) and is associated with pain severity in irritable bowel disease (Qin et al., 2014). However, this is the first report of GPER stimulation having a functional molecular consequence in STS activity.

Intriguingly, E_2_ and G1, a specific GPER agonist, increased STS activity in HCT116 cell lines (**Figure 7**), suggesting a potential novel positive estrogen feedback loop is present within CRC. In theory, greater STS activity should result in increased local E_1_ and E_2_ synthesis. Little is known about the regulation of STS activity. STS can undergo various post-translational modifications resulting in greater activity (Stengel et al., 2008), and this effect may be NF-κB regulated (Jiang et al., 2016). However, there are no other reports that estrogen availability impacts STS activity. In breast cancer, GPER stimulation by tamoxifen does elevate the expression of aromatase (Catalano et al., 2014), the enzyme involved in estrogen synthesis from androgen pre-cursors. It is with some interest then to see that tamoxifen also increased STS activity in HCT116 cell lines. As a selective ER modulator, tamoxifen is a first line therapy against hormone-dependent breast cancer. However, it has recently been shown that tamoxifen may act as a GPER agonist in tamoxifen-resistant tumors (Mo et al., 2013). Thus, tamoxifen-induced increase of STS activity, and therefore increasing local estrogen availability, in CRC and potentially other malignancies may represent a novel GPER-stimulated pathway regulating STS action. It will be of importance to further examine whether tamoxifen and fulvestrant induce STS activity via GPER stimulation, as this may represent a novel route for tamoxifen and fulvestrant resistance.

## Conclusion

We have demonstrated here that CRC cell lines can transport E_1_S and have sufficient STS activity to liberate E_1_. STS activity is possibly regulated by local estrogen availability through GPER stimulation, and this represents a novel positive estrogen feedback loop within CRC. These results have direct consequences for HRT therapy, suggesting that HRT may increase STS activity in the colon leading to potentially undesired effects through GPER action.

## Author Contributions

LG performed qRT-PCR and analysis on cell lines and wrote the manuscript, AG and VT performed STS activity assays, MH performed qRT-PCR analysis and ran the E1S uptake studies, A-MH performed qRT-PCR on cell lines, AA, performed immunoblotting, PF performed E1S uptake studies, STS activity assays, analyzed data, and wrote the manuscript.

## Conflict of Interest Statement

The authors declare that the research was conducted in the absence of any commercial or financial relationships that could be construed as a potential conflict of interest.

## Footnotes

1 https://discover.nci.nih.gov/cellminer/

## Abbreviations

BPS: bromsulphthalein
CRC: colorectal cancer
E_1_: estrone
E_2_: estradiol
E_1_S: estrone sulfate
ER: estrogen receptor
FBS: fetal bovine serum
GPER: G-protein coupled estrogen receptor
HRT: hormone replacement therapy
OATP: organic anion transporter polypeptides
sFBS: charcoal-stripped fetal bovine serum
SLCO: solute carrier organic transporter family
STS: steroid sulfatase
SULT: sulfotransferase
