# Supplementary figures and images for: Estrone Sulfate Transport and Steroid Sulfatase Activity in Colorectal Cancer: Implications for Hormone Replacement Therapy
Source: Front Pharmacol. 2017 Mar 7;8:103. doi: 10.3389/fphar.2017.00103 (PMC5339229; doi:10.3389/fphar.2017.00103)

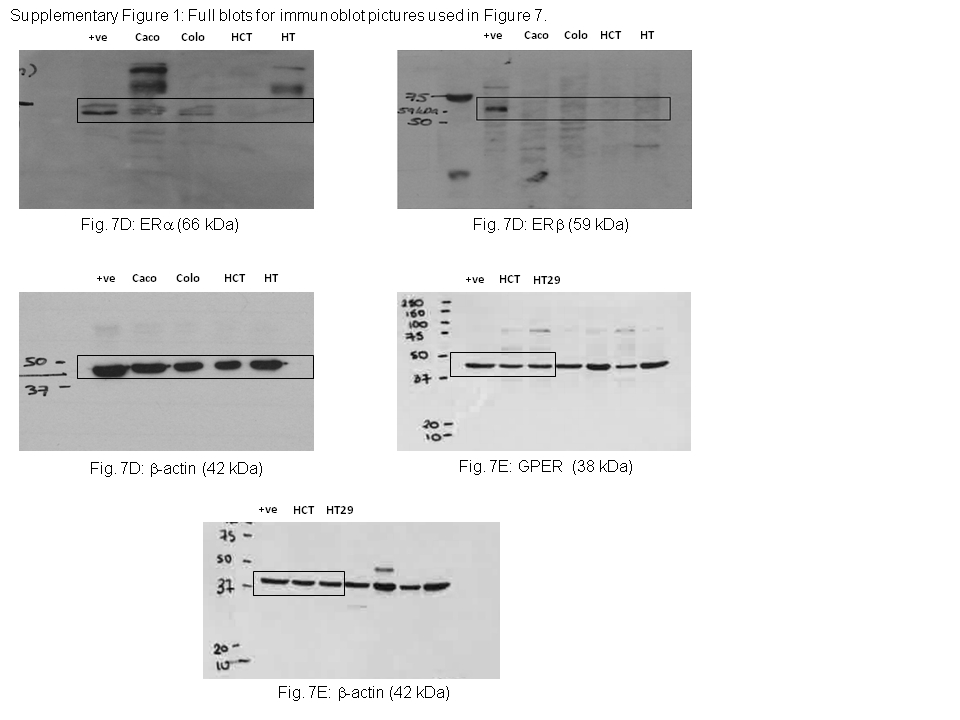

Supplement: Supplementary file 1 [file Image_1.TIF]
